# Pingers are effective in reducing net entanglement of river dolphins

**DOI:** 10.1038/s41598-022-12670-y

**Published:** 2022-06-07

**Authors:** Vishnupriya Kolipakam, Merin Jacob, Aaranya Gayathri, Sunny Deori, Hiyashri Sarma, Syeda Tabassum Tasfia, Anurag Rokade, Ranjana Negi, Abdul Wakid, Qamar Qureshi

**Affiliations:** Wildlife Institute of India, Chandrabani, Dehradun, Uttarakhand 248001 India

**Keywords:** Ecology, Behavioural ecology, Biodiversity, Conservation biology

## Abstract

Ganges River dolphins echolocate, but this mechanism is inadequate for poor sonar-echoing objects such as the monofilament gillnets, causing considerable net entanglement related mortalities. Net entanglement related deaths are one of the major causes of cetacean population decline around the world. Experiments were carried out to understand the use of pingers—an acoustic deterrent, in aiding the deterrence of dolphins from fishing nets. Based on the dolphin clicks recorded, in an experimental setup spanning 36 days, a 90% deterrence was found; 22.87 ± 0.71 SE dolphin detection positive minutes per hour near non-pingered nets versus 2.20 ± 0.33 SE per hour near pingered net. Within 30 m radii of nets, visual encounters of non-calf reduced by 52% and calf by 9%, in the presence of pingers. No evidence of habituation to pingers, habitat avoidance in dolphins after pinger removal or a change in fish catch in nets because of pingers was found during the study. While the effectiveness of pingers on calves and fish catch needs further experimentation, the use of pingers to minimize net entanglement mortalities in the Ganges River dolphins seems to be the most promising solution currently available. These results have critical implications for the conservation of other species of river dolphins around the world.

## Introduction

The Ganges River dolphins (*Platanista gangetica)* are endangered freshwater cetaceans that have adapted to inhabit the turbid waters of the Ganga–Meghna–Brahmaputra–Karnaphuli–Sangu river systems in the Indian subcontinent^1–3^. As part of this adaptation, Ganges River dolphins have reduced visual abilities^4,5^, allowing them to only perceive directionality of light sources, but not to identify or resolve objects underwater^6^. Therefore, they depend on their echolocation abilities, rather than on visual cues, for most of their activities such as foraging, navigation and intraspecific communication. The echolocation mechanism of these dolphins involves a ceaseless emission and reception of biosonar clicks to recognize the presence of objects underwater^7,8^. While this suffices in most cases, it is inept at recognizing objects such as the monofilament gillnets; monofilament nets, especially those of nylon, have density similar to water and hence give poor sonar echo^9,10^. This has led to a considerable number of dolphin net entanglements that have been lethal in many cases and increased chances of getting bycaught in others^3,11,12^.

The probability of dolphin net entanglements, either in the deployed fishing nets or ghost nets (nets that are discarded or strewn in the river), is currently unknown. However, the river systems that the Ganges River dolphins inhabit record a considerable intensity of fishing activities, especially of the monofilament gillnets. From the surveys conducted, active gillnet fishing averages at least one net every 2 km in the Brahmaputra mainstream and one net every 3 km in the Ganga mainstream^13^. Net entanglements may be classified as ‘accidental’ in many cases; however observations from local fishers have revealed that dolphins feed on the fish that are caught in the deployed fishing nets, and this behavior further increases their propensity to become entangled. A dolphin, once entangled, has narrow chances of being set free. A social survey of fishers in West Bengal revealed that 95% of them have stated they sell the dolphins, if and when they are bycaught, for their oil, rather than rescuing and releasing them back^14^. The mortalities that may hence arise from these net entanglements will certainly not be negligible. Reports reveal that out of the 21 known mortalities of dolphins, in Brahmaputra, in 2008 alone, 20 were victims of net entanglement^15^, as were 7 out of the 14 deaths that were recorded between 2018 and 2020 in the rivers of West Bengal (Qureshi et al., unpublished). Given that the species population is decreasing^16^, it is imperative to examine and implement a managerial intervention and ensure minimal net entanglement-related mortalities, to ensure the long-term survival of the species.

Cases of net entanglement mortalities and the need for managerial intervention are not novel to aquatic ecosystems; they span multiple species of cetaceans^17–20^, and many studies have been carried out to identify and adopt methods that can deter animals from deployed fishing gear. Among these, some deterring mechanisms tested for gillnets include using acoustically reflective nets, visually detectable nets, ‘‘buoyless’’ nets, reduced-strength nets, nets with weak links^21–26^ or deploying sound producing devices on nets^26^. Acoustically reflective nets, which involve changing net gear material or adding acoustic reflective materials such as metals to increase the acoustic detectability of the nets, have not been efficient in deterring^27–30^. Alternatively, increasing the visibility of nets by using light-emitting diodes or using particular net rope colours has worked in deterring only some marine cetaceans^31,32^ that are capable of underwater vision. Sound producing devices with low acoustic outputs are effective in deterring small cetaceans, in some species but not in others^32,33^, depending on many factors including aspects like species’ communication^34^ and behavioural differences^17,26^. For deterring a species that relies mostly on echolocation rather than visual cues, such as the Ganges River dolphins, and in habitats where sustenance fishing is prevalent, a deterrent that is acoustic and economically viable, less invasive and easier to adopt is more likely to aid in reducing bycatch mortalities. Among the available options, sound producing devices also known as pingers, would be the best suited for the Ganges River dolphins. The deployment of such a device on fishing nets should, in theory, act as an indication of the presence of an object to river dolphins within a spherical range. This would subsequently act as a warning signal and deter the dolphins from approaching the fishing nets. Pingers as deterrents have been tested for some river dolphins; in Franciscana dolphins, from a 2-year long study, it was reported that the bycatch rate was 10 times lower in nets with pingers than in those without pingers^35^, and an ongoing study on Irrawaddy dolphins reports that the minimum distance of dolphin approach to nets with pingers is 10 m away, unlike those without pingers^36^. A similar trial was carried out for the Ganges River dolphins in one of the Brahmaputra tributaries (Kulsi)^37^. Through visual observation of dolphin surfacing, it was seen that the minimum distance of approach of dolphins to nets shifted from <1 m to >5 m in the presence of pingers on the net^37^. However, as this study did not have supporting acoustic evidence to corroborate these observations, and since efficacy of this method for prolonged use should be established, further research is needed.

While the visual study by Deori et al.^37^ demonstrated evidence of visual deterrence, it is important to assess, in a robust manner, whether pingers can be a viable option for long-term and extensive use. To do so, one must unequivocally establish that there are negligible long-term effects that might lead to increased risk. There is concern that pingers, instead of deterring, tend to function as an unintentional dinner bell and thereby increase entanglement probability^28,38,39^. Acoustic deterrents are sometimes found to deter dolphins permanently, causing loss of habitat available to dolphins^17,40^. Since pingers are acoustic emitters with set acoustic characteristics, the response to pingers might vary with the dolphin age class, as the ability to process the directionality of returning echoes through head scanning develops over age after a postnatal period, suggesting a weaker pinger response in calves compared to non-calves^40^. Using pingers might also cause a change in the dolphin vocalization patterns, as dolphins have shown a masking behavior, i.e., altering their acoustic characteristics in the presence of anthropogenic noise^41^. Similarly, fish have also been reported to become acoustically deterred by pingers^42,43^, thereby reducing their usability by fishers. Considering these potential impacts, in our current study, we address the important questions by (a) quantifying the efficacy of pingers as a deterrent and the response of different age classes of dolphins, (b) assessing the possibility of detrimental effects such as long-term area avoidance or change in vocalization patterns and (c) assessing the effect on fish catch.

## Study area, materials and methods

**Figure 1:**
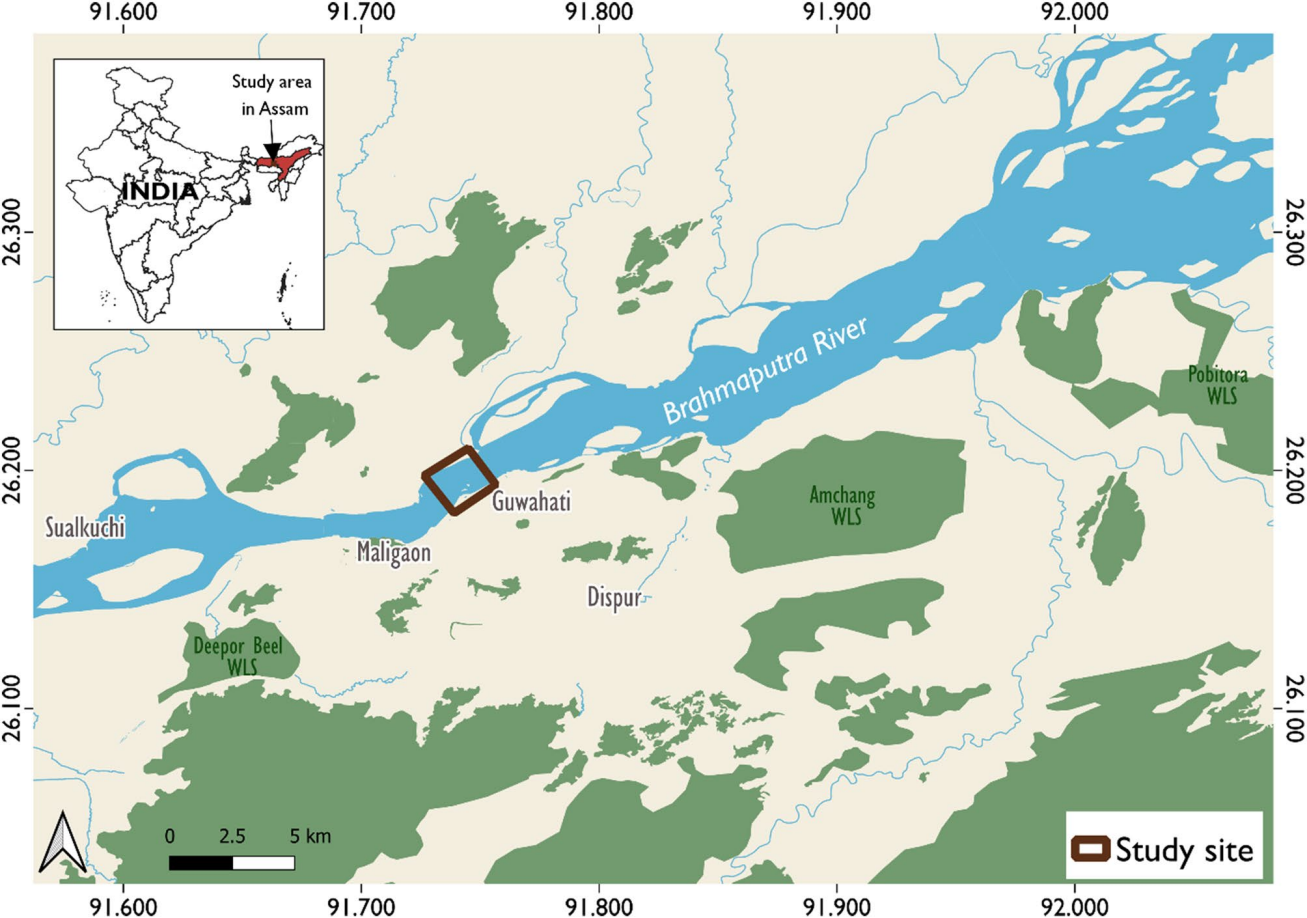
Study area map showing the site for pinger experiment near Guwahati (Inset: State map of India with Assam State highlighted), generated using QuickOSM plugin for QGIS, Version 1.16.0 (https://github.com/3liz/QuickOSM), and visualized through QGIS software version 3.8.2.

The study was carried out in a stretch of the river along Guwahati, a part of the Brahmaputra mainstream in the Assam State of India (Fig. 1). The site was chosen after a preliminary survey, ensuring that there is a presence of a resident population of at least 10 dolphins within a 10 km radius, including calves. The project was accorded appropriate permissions by the MoEFCC and Forest Department of Assam, who regulate research permissions, keeping in view the status of the species. The study was carried out in accordance with relevant guidelines and regulations.

**Figure 2:**
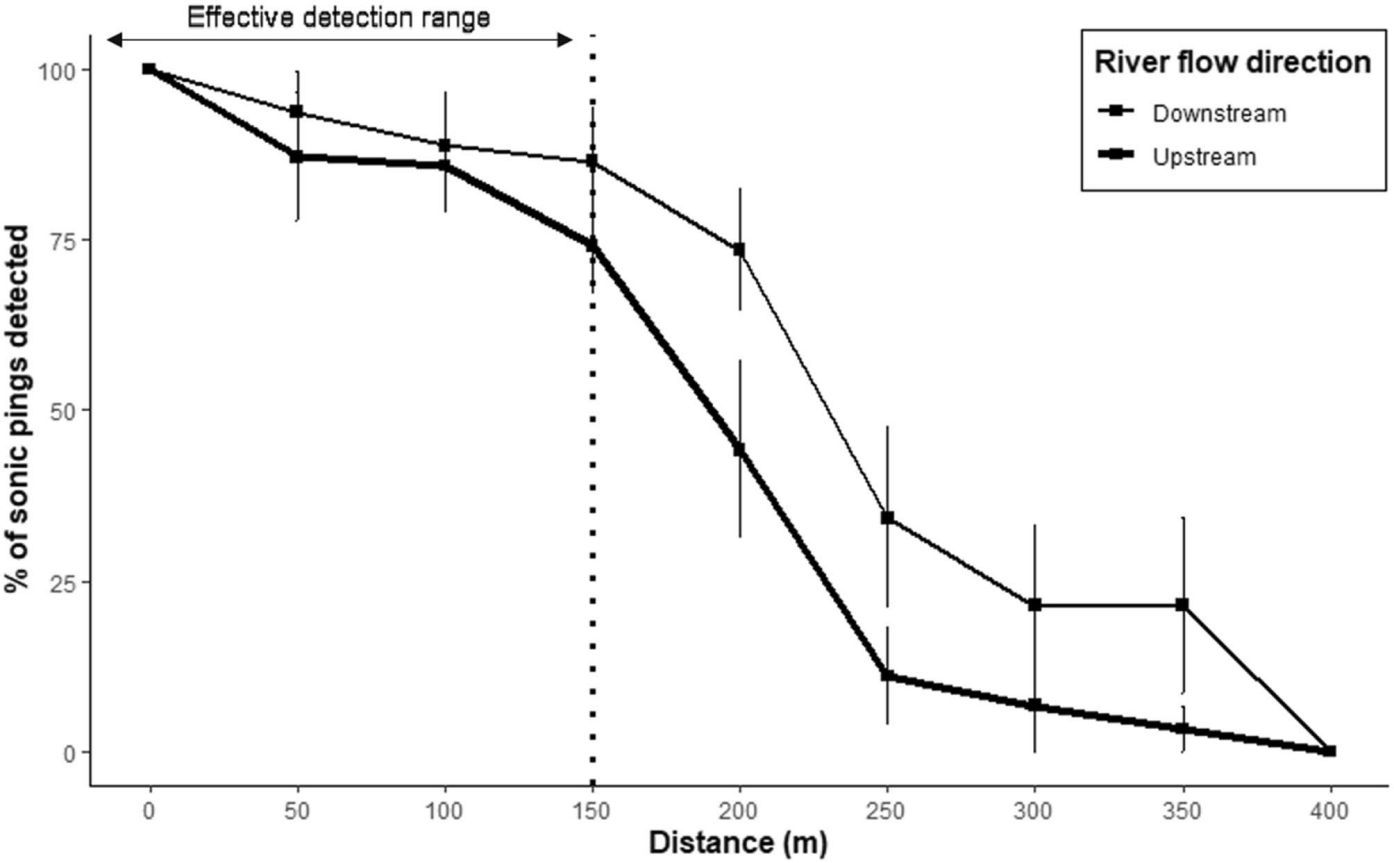
Effective range of pingers. Until 150 m, the average % of sonic pings detected in the CPOD is 89.5 ± 2.4 SE and the average beyond 150 m is 24.6 ± 4.4 SE.

Pingers are acoustic emitters that produce sonar sounds at regular intervals. In the current study, pingers used were manufactured by Future Oceans Inc and were fabricated to ensure that sonic pings fall within the acoustic range of the Ganges River dolphins. These pingers produce sonic pings of a frequency of 70 kHz at a sound pressure level or loudness of 175 dB (Netshield Dolphin Anti-Depredation Pinger) with an inter-click interval of 4 ms. The effective range of the pinger was determined by deploying it at 50 m intervals for a distance of 500 m from a passive acoustic monitoring device (CPOD), and plotting a distance decay graph of the number of sonic pings recorded. CPOD records and logs acoustic data that fall within the sound spectrum of the CPOD’s hydrophones. Based on the percentage of sonic pings detected, the effective range was determined to be 150 m (Fig. 2), where the average detection was more than 75% both upstream and downstream of the CPOD.

We carried out one experiment to assess the effect of pingers on dolphins and another experiment to understand its effect on fish catch.

### Experiment 1

In this experiment, the efficacy of pingers, the response of different age classes of dolphins, and the effect of prolonged use of pingers on dolphin habitat use and acoustic characteristics were quantified. For the effects of long-term use, two alternative scenarios were hypothesized: (a) dolphins will actively avoid the area post-removal of pingers, i.e., loss of available habitat for dolphins, and (b) dolphins will continue using the area post-removal of pingers, i.e., the presence of pingers does not deter future use of habitat. This aspect is extremely crucial to understand if pingers are to be proposed for widespread use to ensure that an extensive use does not lead to habitat loss for the dolphins. We also tested whether the use of these acoustic deterrents has an effect on dolphin acoustic characteristics.

**Figure 3:**
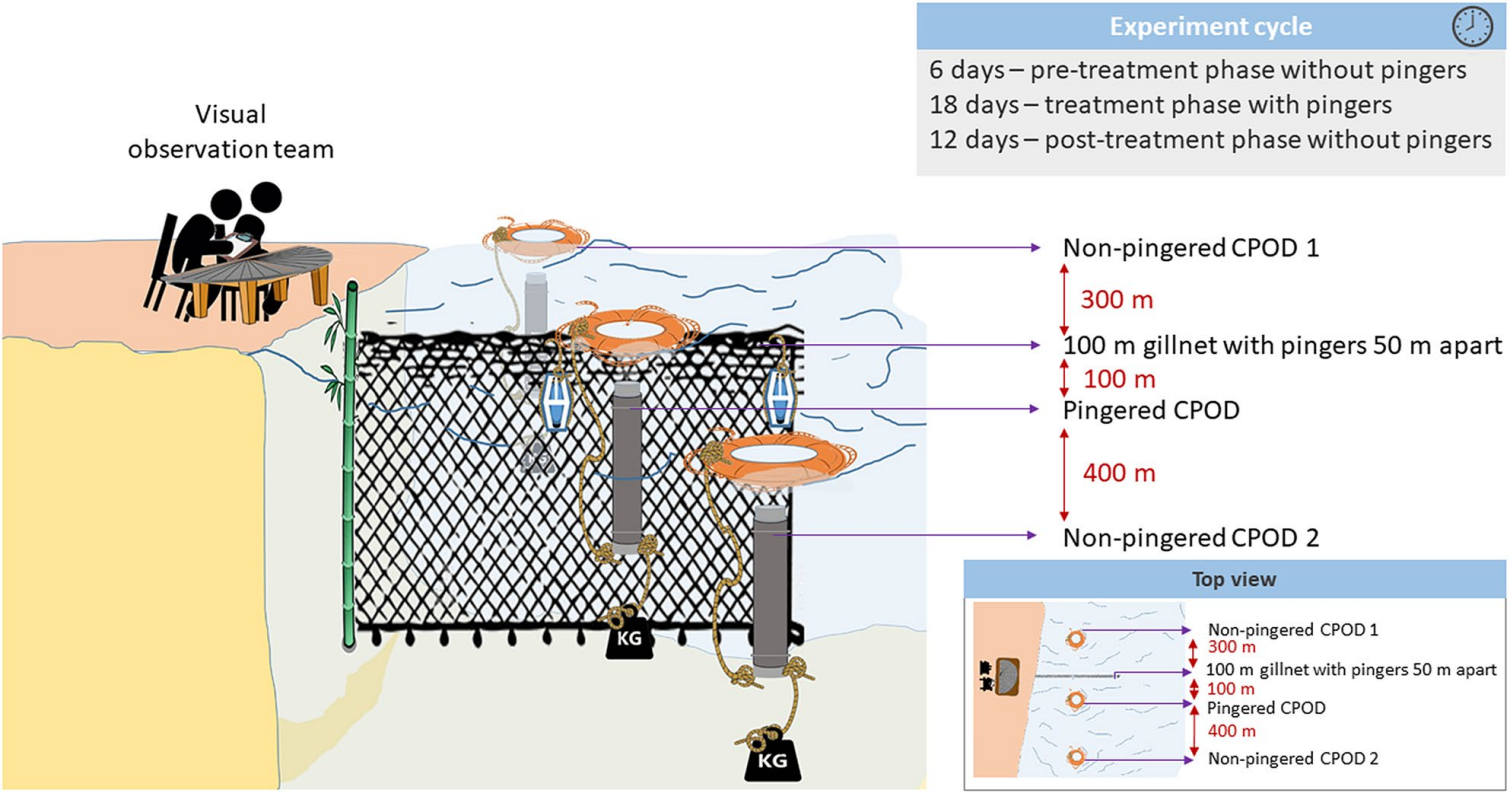
Experiment set up depicting the placement of CPODs, the net with pingers, visual team and the details of different phases of the experiment cycle.

To address the above three objectives, a control-treatment experiment was set up in a stretch of Brahmaputra in Guwahati, Assam, during December 2020–March 2021. The fishing net deployed was in accordance with the practices of the local fishers for that particular season; in this case, it was a nylon bottom gillnet of mesh size 15 mm and a length of 100 m. The pingers deployed were at a regular interval of 50 m along the length of the net. A CPOD was deployed parallel to the net, hereinafter referred to as the pingered CPOD. This CPOD was deployed 100 m away from the centre of the net in order to avoid logging of loud sonic pings that might mask the clicks of the Ganges River dolphins around it. The CPOD was kept active for 24 h a day until the experiment was completed. The controls used were spatial and temporal to address the different objectives (experimental setup in Fig. 3). There were two control CPODs 400 m away (one upstream and one downstream) from the pingered net, where the effect of pingers is near negligible, hereinafter referred to as non-pingered CPODs. These two CPODs were active 24 h a day until the experiment was completed. It was hypothesized that during the active phase of pingers on the nets, there would be a reduction in the dolphin net visitation rate and therefore a reduction in the acoustic recording of dolphins on the pingered CPOD. This is substantiated by comparing the data with non-pingered CPODs, to eliminate the influence of variation brought about due to space-use by dolphins. This would serve to determine the efficacy of pingers in deterring dolphins from the pingered net. The experiment began with of a ‘*pre-treatment*’ phase for 6 days, where the CPODs and net were in their respective positions, but the pingers were not deployed. This was followed by a ‘*treatment*’ phase for 18 days, where the pingers were active. Subsequently, the pingers were removed and CPODs monitored for dolphin presence in the ‘*post-treatment*’ phase of 12 days.

### Acoustic data

Dolphin presence was quantified by extracting the “Detection Positive Minutes” per hour (hereinafter as DPM/h) from the CPOD. DPM/h indicates the number of minutes with at least one dolphin click recorded in an hour in the CPOD. As the Ganges River dolphins ceaselessly produce biosonar clicks^7,8^, the DPM/h would be directly indicative of the dolphin presence within the effective range of the CPOD^44^. For the acoustic parameters, we extracted the most dominant frequency, sound pressure level (or loudness—SPL) and inter-click interval (ICI) of all the dolphin clicks recorded in the CPOD. All the CPOD data were extracted using the CPOD.exe software once the experiment was completed and the CPODs were retrieved. During data extraction, clicks classified by the CPOD software as "other cetaceans" in "high" and "moderate" quality (an index of the accuracy of classification; CPOD manual by Chelonia Ltd.) were only used.

### Visual data

A visual observation team consisting of one observer and one recorder were stationed on the bank at the site of deployment of fishing net and recorded dolphin surfacing from 0930 to 1530 h throughout the treatment and post-treatment phases, summing to 25 days. The team, however, was not aware of the experimental phase, i.e., whether fishing nets contained pingers or not. For each sighting of a dolphin surfacing, the observer provided the recorder with the time, angle of surfacing, estimated distance to the dolphin and age class (calf, non-calf or unknown). The compass bearing of the visual observation direction was also recorded from the observation location. The locations of the net and the pingers were also geo-mapped. These details were used to project and determine the GPS locations of all the dolphin-surfacing recordings and thereby to calculate the distance of dolphin approach to the fishing net.

### Analytical methods

Data was analysed in Excel and RStudio using the *Base* package and visualizations were performed using *ggplot2::ggplot*^45^ in RStudio vers 1.4.1717^46^.

### Efficacy of pingers in deterring dolphins

To establish that the control/non-pingered sites and treatment/pingered sites have comparable dolphin activity, acoustic data (DPM/h) were compared, using ANOVA’s, during the pre-treatment phase from all the three deployed CPODs. To determine the efficacy of pingers as deterrents (a) non-pingered CPODs and pingered CPOD during the treatment phase were compared^47^, and (b) pingered CPOD during the pre-treatment and treatment phases were compared, using ANOVA and Tukey’s HSD. This data was corroborated using Kolmogorov-Smirnov tests on visual observation^47^. The reduction in DPM/h at the pingered CPOD or approach distance to the net during the treatment phase demonstrates the efficacy of pingers as deterrents.

### Effect of extensive use of pingers on habitat use and acoustic characteristics of dolphins

Sustained deterrence from an area might lead to long-term avoidance of habitat by dolphins. In such a scenario, widespread use of pingers would mean a reduction in habitat available for dolphins. To understand if there is such an effect, the DPM/h at pingered CPOD during the pre-treatment and post-treatment phases was compared using ANOVA and Tukey’s HSD.

Similarly, since the deterrent used is acoustic, there is a possibility of an acoustic response of dolphins to the presence of these pingers. We assessed whether there was a change in acoustic characterization of dolphin vocalization during the deployment of pingers, and whether this change persisted after the removal of pingers. The acoustic characteristics—SPL, frequency and ICI of dolphins during pre-treatment, treatment and post-treatment phases at the pingered CPOD—were compared using chi-squared test^47^. We also assessed if there is an increased or decreased usage of a particular frequency, SPL or ICI range, especially around the characteristics of the pinger used—70 kHz frequency, 175 dB SPL and 4 ms ICI. We calculated the proportion of usage of frequency, SPL and ICI in each 10-unit bin, i.e., the proportion of usage, for example, in 10–20 kHz, 21–30 kHz frequency bin, for each of the three experiment phases recorded at the pingered CPOD. We located the change by doing a pair-wise comparison between the three experiment phases by comparing the values of the effect sizes in each of these 10-unit bins of frequency, SPL and inter-click interval. Effect size being a direct measure of the magnitude of difference between two groups, values are directly indicative with larger effect size indicating higher differences.

### Response of different age-classes of dolphins to pingers

To understand the response of different age class dolphins to pingers, we compared their approach distances during the presence and absence of pingers, using chi-squared test.

### Experiment 2

To understand the effect of pingers on fish catch, we measured the fish catch from the pingered net laid for Experiment 1, for 10 consecutive days (5 days each during pingered and non-pingered phases). We used a temporal control-treatment set up to address this objective, where fish catch was measured during the time when pingers were deployed on the net, and compared with the fish catch after removal of pingers. In this setup, the pingered phase preceded the non-pingered phase, in order to tease apart the effect of pingers and harvesting on depletion. To quantify the fish catch, the net was retrieved 2–3 times a day, the fish catch collected, and net redeployed. From the fish catch, the species caught and the total weight of fish obtained were recorded along with the fishing effort in hours. From these, catch per unit effort (CPUE) was calculated during both phases. We compared the CPUE between the two phases using a t-test.

## Results

### Experiment 1

#### Efficacy of pingers in deterring dolphins

**Figure 4:**
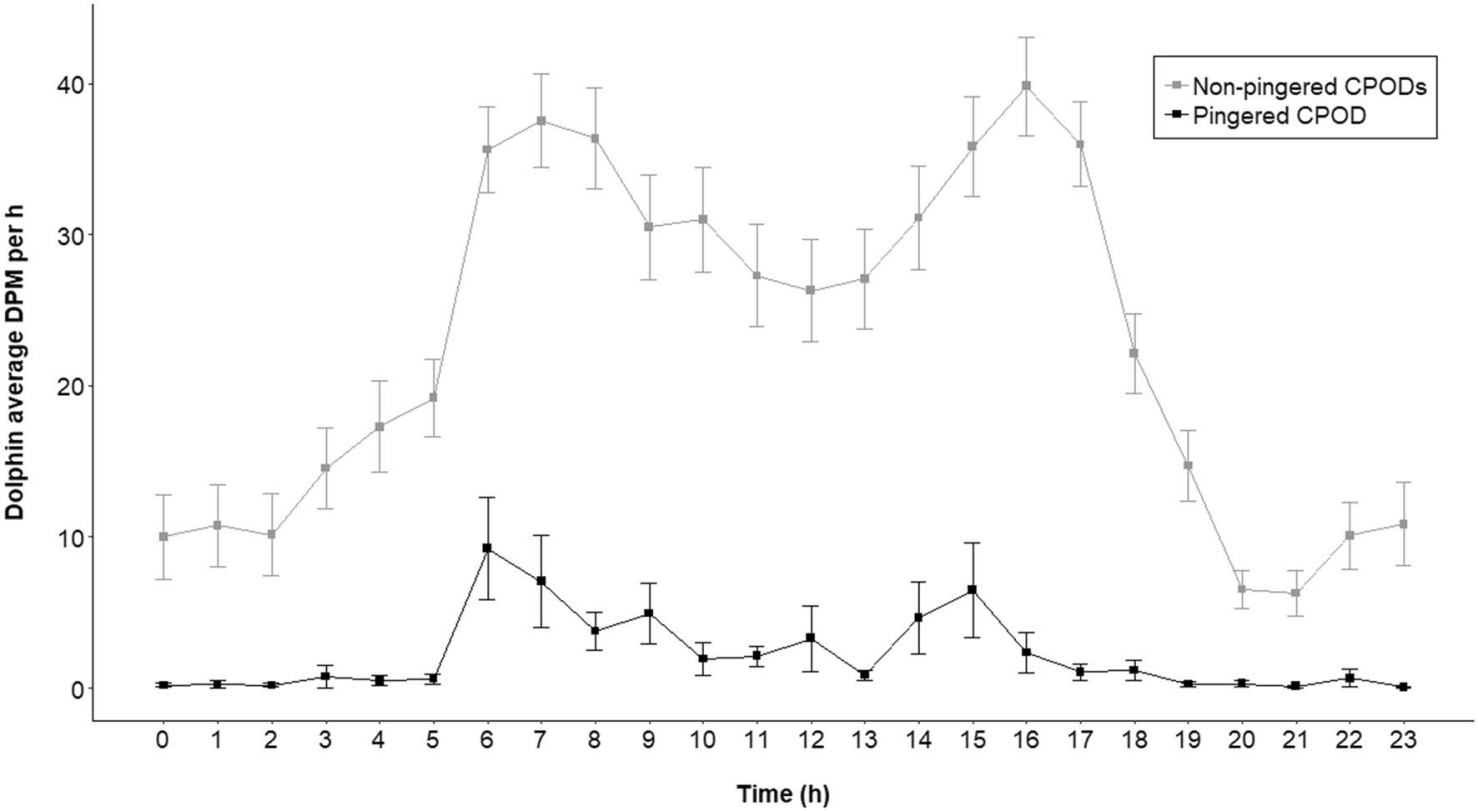
Average dolphin presence recorded in a day in the pingered and non-pingered CPODs during the treatment phase of 18 days when pingers were active.

**Figure 5:**
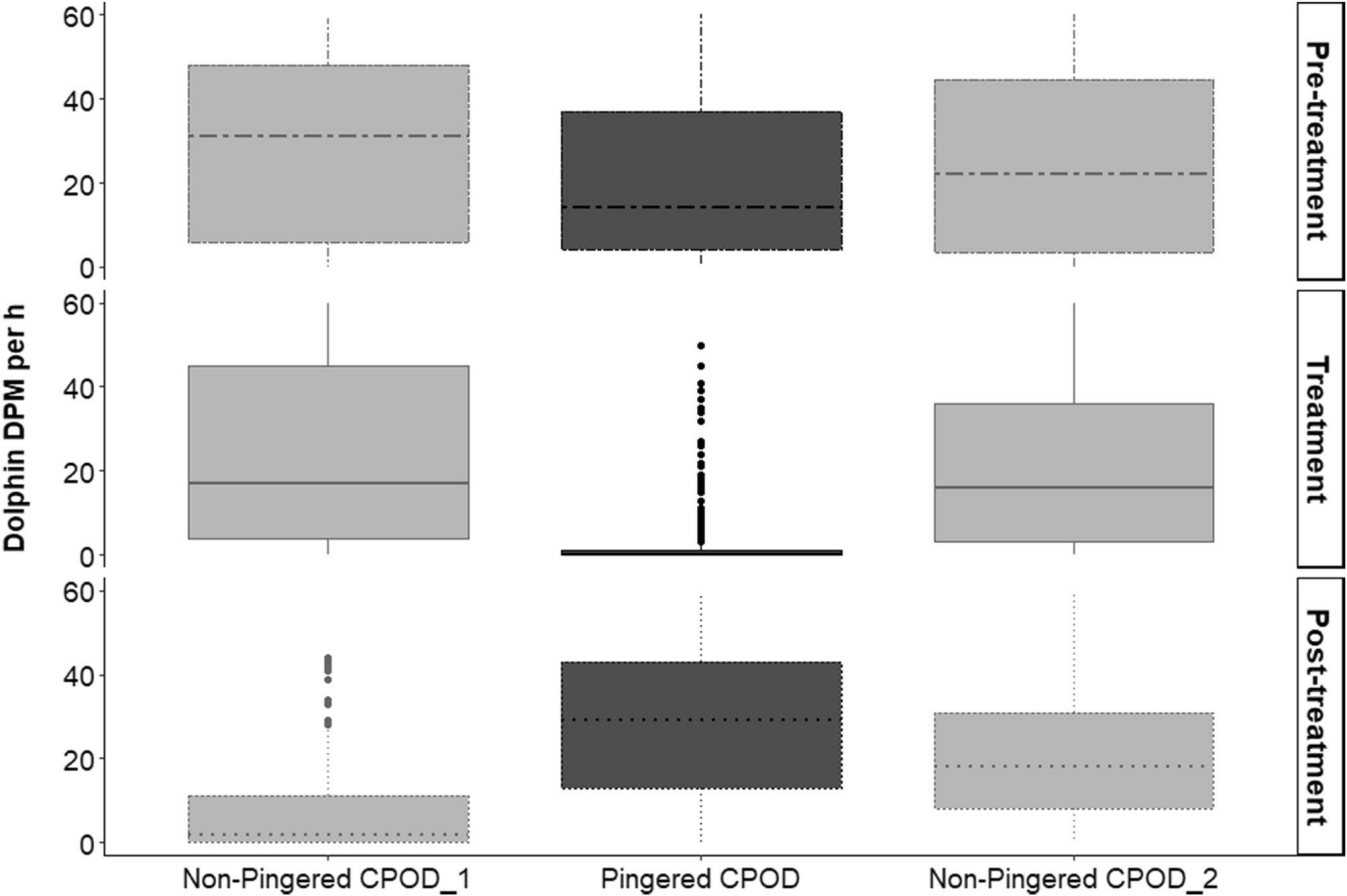
Dolphin presence recorded in the three CPOD sites during the pre-treatment, treatment and post-treatment phases.

The pre-treatment phase of 6 days yielded 109 h of acoustic data. There was no difference in dolphin presence (DPM/h) in the three deployed CPOD sites (ANOVA F = 0.842, p = 0.435), indicating that the three sites were comparable before treatment. The treatment phase of 18 days yielded 414 h of acoustic data in the pingered and the non-pingered CPODs each. There was a significant difference in the dolphin presence among the three sites (ANOVA F = 37.24, p < 0.0001). The non-pingered CPOD recorded an average DPM/h of 22.87 ± 0.71 SE per h, while the pingered CPODs recorded an average DPM/h 2.20 ± 0.33 SE per h (Tukey's HSD p < 0.001) (Fig. 4). The dolphin presence in the pingered CPOD was significantly lower during the treatment phase, than during the pre-treatment phase (ANOVA F = 39.92, p = 3.03e−12; Tukey's HSD p < 0.001) (Fig. 5), indicating that pingers effectively deterred the dolphins.

The visual observation yielded 112 h of data of dolphin surfacing collected over a period of 25 days with 4800 encounters of dolphin surfacing within 700 m radii of the observation team. The visual data on surfacing dolphins showed that, within 30 m from the net, there was a reduction of almost 50% of dolphin surfacings during pingered phase (14.76% surfacing), when compared to the non-pingered phase (27.74%) (KS test D = 0.9, p < 0.001).

#### Long-term effect of deterrent usage on habitat use by dolphins

**Figure 6:**
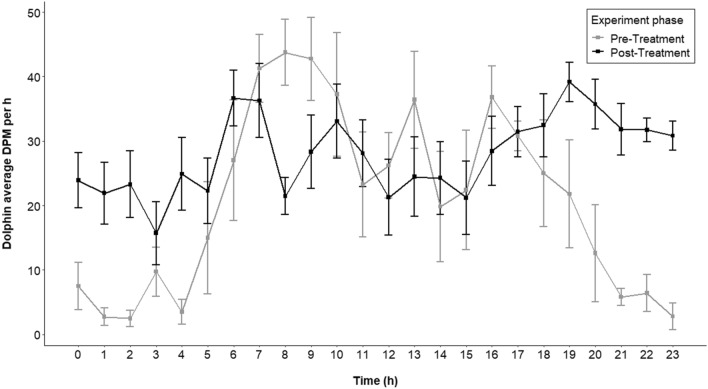
Average dolphin presence recorded in a day in the pingered CPOD during the pre-treatment and post-treatment phase of 6 and 12 days, respectively.

The post-treatment phase of 12 days yielded 263 h of acoustic data in the pingered and the non-pingered CPODs each. There was no difference in the average dolphin DPM/h recorded during the pre-treatment phase (20.91 ± 1.82 SE per h) and post-treatment phase (27.89 ± 1.04 SE per h; Tukey’s HSD p = 0.42), indicating that the dolphin presence recovers upon removal of pingers and that the deterrence is not persistent (Figs. 5 and 6).

#### Dolphin acoustic behaviour during the presence and absence of acoustic deterrent device

##### Modal frequency

**Figure 7:**
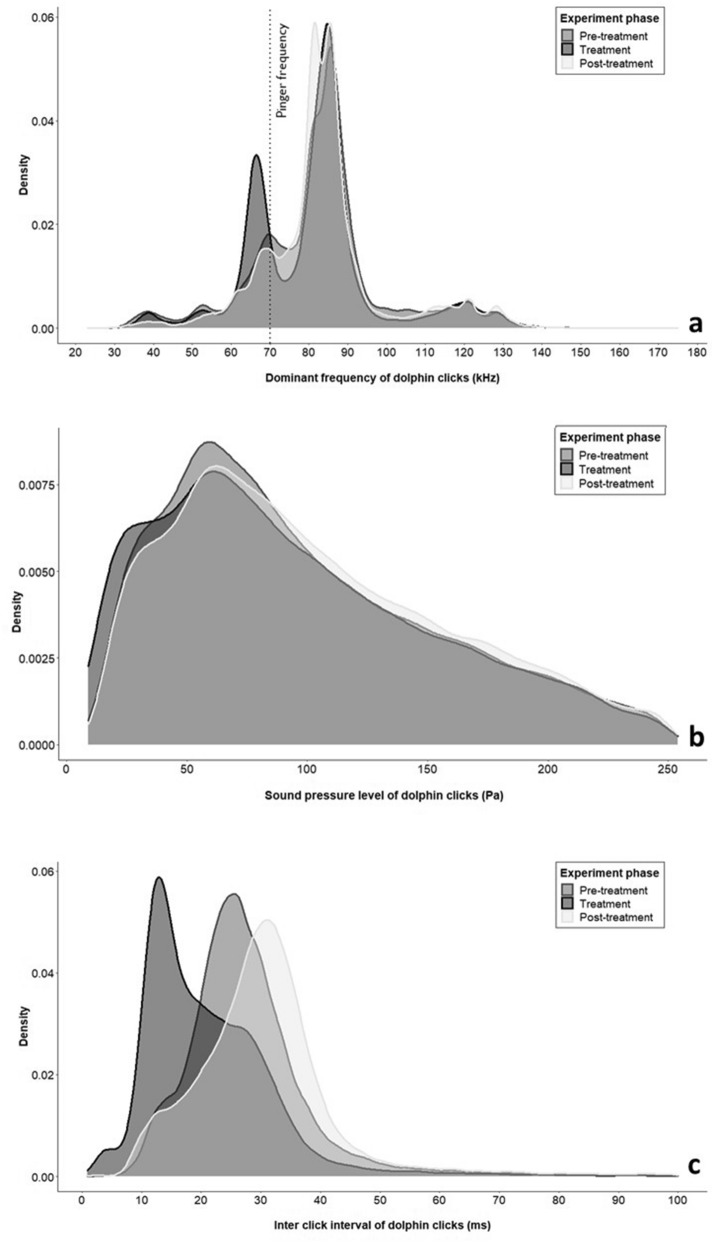
(**a**) Density plot of dominant frequency of dolphin clicks (kHz) during the three phases of the experiment, (**b**) Density plot of sound pressure level of dolphin clicks (Pa) during the three phases of the experiment and (**c**) Density plot of inter-click intervals of dolphin clicks (ms) during the three phases of the experiment.

The most dominant frequency used by the dolphin was 82.52 ± 0.09 SE kHz during the pre-treatment, 81.17 ± 0.10 SE kHz during the treatment and 83.73 ± 0.06 SE kHz during the post-treatment phases. There was no significant difference found in the modal frequency used by dolphins during the pre-treatment and post-treatment phases (chisq value = 16.09, p = 0.13, df = 11). However, there was a significant difference between the pre-treatment and treatment phases as well as between the treatment and post-treatment phases (pre-treatment and treatment: chisq value = 56.11, p < 0.001, df = 11; treatment and post-treatment: chisq value = 65.94, p < 0.001, df = 11) (Fig. 7a). Effect size ranged from 0 to 6.00 for all frequency bins between pre-treatment and post-treatment phases, however, on comparing the pre-treatment and post-treatment with the treatment phase, the effect size ranged from 0 to 6.75 for all the frequency bins except a peak in the 60–80 kHz bin (effect size range: 9.44 to 17.93) (Supplementary Figure S1). This marginal shift of dolphin frequency usage in the 60–80 kHz bin during the treatment phase also happens to be the frequency of the pinger, and this shift reverts on removal of pingers.

##### Sound pressure level

The sound pressure level (i.e., loudness) used was 98.33 ± 0.31 SE Pa during the pre-treatment, 95.27 ± 0.35 SE Pa during the treatment and 101.99 ± 0.22 SE Pa during the post-treatment phases. The sound pressure level did not differ significantly in the three phases of the experiment (pre-treatment and post-treatment: chisq value = 3.65, p = 1, df = 23; pre-treatment and treatment: chisq value = 15.27, p = 0.88, df = 23; treatment and post-treatment: chisq value = 15.93, p = 0.85, df = 23) (Fig. 7b). The effect size ranged from 0 to 3.01 for all the SPL bins (Supplementary Figure S2).

##### Inter-click interval

The inter click interval (ICI) which is the time difference between two clicks within a dolphin train was found to be 27.88 ± 0.06 SE ms during the pre-treatment, 21.24 ± 0.06 SE ms during the treatment and 30.58 ± 0.04 SE ms during the post-treatment phases. There was a significant difference in the dolphin inter-click interval in the three phases of the experiment (pre-treatment and post-treatment: chisq value = 76.43, p < 0.001, df = 9; pre-treatment and treatment: chisq value = 276.01, p < 0.001, df = 9; treatment and post-treatment: chisq value = 376, p < 0.001, df = 9) (Fig. 7c), indicating a shift in the dolphin inter-click interval (Supplementary Figure S3). The effect size ranged from 1.16 to 23.43 for all the bins except a peak in the 10–20 ms (effect size: 35.92) in the presence of pingers. On removal of pingers, the effect size ranged from 2.97 to 16.12 for all the bins except for a peak in the 10–20 ms and 30–40 ms (effect size: 41.48 and 34.99).

#### Response of dolphins of different age classes to pingers

**Figure 8:**
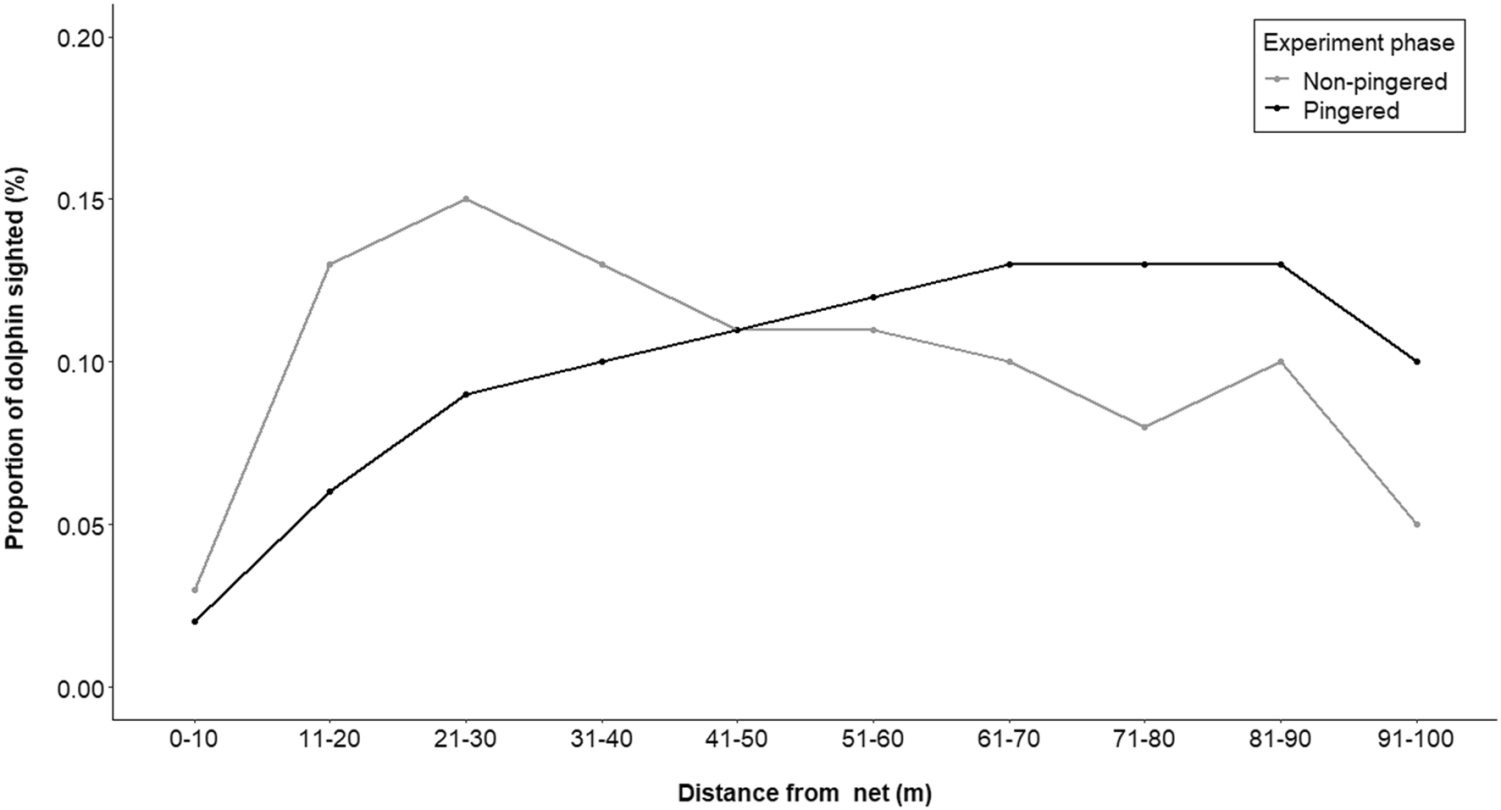
Proportion of non-calves sighted by visual observers during the pingered and non-pingered phases of the experiment at different distance classes.

**Figure 9:**
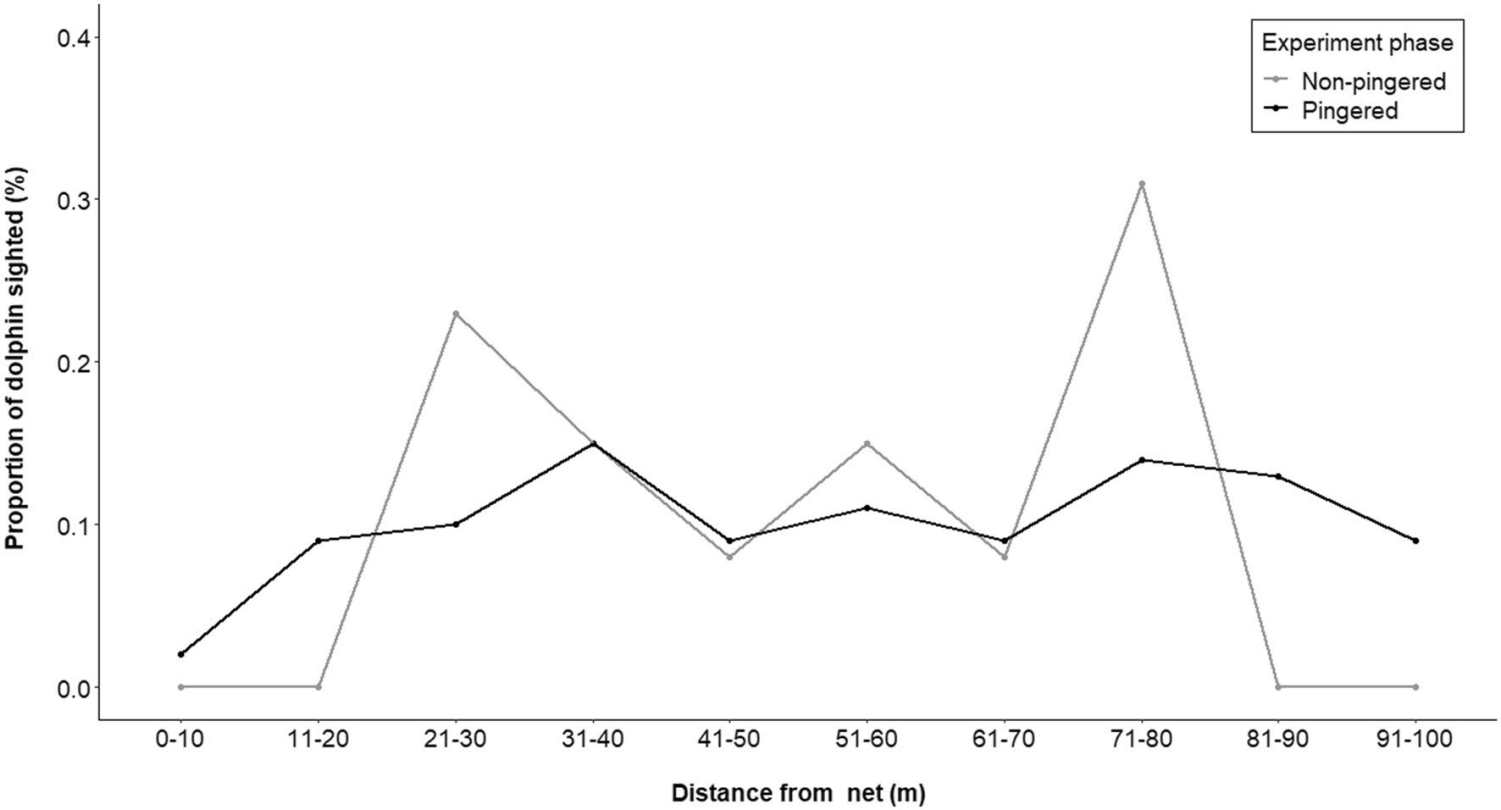
Proportion of calves sighted by visual observers during the pingered and non-pingered phases of the experiment at different distance classes.

The visual data of 112 h of dolphin surfacing encompasses 4800 dolphin surfacings (3029 non-calves, 613 calves). In the 30 m zone from the fishing net, the total percentages of non-calf and calf sightings were 31% and 23%, respectively during the non-pingered phase. However, within the same zone during the pingered phase, the average percentages of non-calf and calf sightings were 16% and 21%, respectively. This indicates a 52% significant reduction (chisq value = 66.46, df = 10, p < 0.05) in the non-calf surfacings within a 30 m zone from pingered-nets (Fig. 8). However, for the calves, while there was a 9% reduction within the 30 m zone, there was no significant difference between the pingered and non-pingered phases (chisq value = 9.19, df = 9, p = 0.42) (Fig. 9).

### Experiment 2

#### Effect of pingers on fish catch in nets

An average of 116 ± 32.19 SE kg of fish catch was obtained in the pingered phase, and 160 ± 29.66 SE kg in the non-pingered phase. Four species of fish were captured during the pingered phase, viz., *Crossocheilus latius*, *Gagata cenia, Botia dario* and *Johnius coitor, * while one species of fish was captured during the non-pingered phase, viz., *Gagata cenia*. There was no significant difference in the average CPUE of fish during the pingered phase with 0.003 ± 0.02 SE fish per unit hour and non-pingered phase with 0.0024 ± 0.00 SE fish per unit hour (t-test p = 0.14, df = 8).

## Discussion

Net entanglement related mortalities are one of the major threats for both marine and freshwater cetaceans, prominently the incidental catch or bycatch of cetaceans in fishing gear^2,14,48–52^. In recent times, bycatch mortality due to fishing gear has led to the extinction of cetaceans such as Vaquita (*Phocoena sinus*)^53,54^ and the Yangtze River dolphin (*Lipotes vexillifer*)^55^. In the case of riverine dolphins and other dolphins, the risk of bycatch is particularly high, as preferred dolphin habitats coincide with prime fishing areas, especially those using gill nets. Furthermore, no-fishing regions are sparse unlike the marine system^56–60^, thus implementation of successful bycatch mitigation measures in freshwater ecosystems are imperative for the conservation of river dolphins.

This study assesses the efficacy and usability of employing acoustic deterrents such as pingers to reduce bycatch mortality of the endangered Ganges River dolphins. Pingers have been demonstrated to effectively decrease cetacean presence around pingered nets (harbour porpoises^43,61^, Franciscana dolphins^35^, short‐beaked common dolphins^62^, beaked whales^34^, narrow-ridged finless porpoises^63^, Burmeister's porpoise^64^). Our study demonstrates an 85 to 95% decline in detection positive minutes of dolphin clicks with the use of pingers (Fig. 5). For a continuous emitter such as the Ganges River dolphins, this indicates an avoidance of the area when pingers are deployed on the fishing nets, which was corroborated by our visual experiment in which we see dolphin surfacing decrease by half within 30 m from the fishing net. This demonstrates that the pingers are successful in deterring dolphins, thereby minimizing the risk of entanglement and bycatch (Fig. 4).

While it is encouraging to see the effectiveness of pingers in reducing the proximity of dolphins to nets (Figs. 4, 5, 8), there are several caveats to understand before putting them into widespread use^65^. For a species that relies on acoustics for majority of its life history functions, the underwater soundscape is a crucial element. The continuous emittance of pings by acoustic deterrents are likely to affect the underwater soundscape and thereby affect dolphin vocalization. A study assessing the response of vessel noise on the Ganges River dolphin’s vocalization patterns showed that some of the acoustic parameters, namely, train duration, clicks per train and frequency range increased in the presence of vessel noise^41^. In our experiments, the acoustic characteristics of the Ganges River dolphin clicks were similar across the three phases (Fig. 7) except for an evident shift in the usage of frequency spectrum (Fig. 7a) and the inter-click interval (Fig. 7c). It is evident that this observed peak in frequency overlaps with the frequency of the pinger used—a masking behavior similar to what was seen in the Ganges River dolphins’ response to vessel noises^41^. However, this behavior of masking is not seen post pinger removal (Fig. 7), supporting that the effect of pingers on the dolphins is only temporary. However, it is still unclear what causes the peak in the inter-click intervals in specific bins and whether it is due to the differential use exhibited by the dolphins of different age-classes as reported by Sugimatsu et al.^66^. Visual data did reveal that pingers were more effective in deterring non-calves (Fig. 8) than the calves (Fig. 9) suggesting a differential response to pingers. It is already understood that the ability to determine the directionality of returning echoes develops in some cetaceans with age. Future studies should explore this aspect.

As mentioned earlier, it is important to understand the risk of using pingers, incase they either act as attractants, increasing the risk of entanglement, or permanent deterrents, thereby causing loss of available habitat for dolphins. Pingered nets have increased bycatch rates in many pinnipeds like the sea lions and cetaceans like the bottlenose dolphins due to increased depredation from the “dinner-bell” effect of pingers^67–69^. Use of pingers have caused exclusion of important habitats for some species like the harbour porpoises, due to the pinger sounds^70^. In some cases, pingers have lost their efficacy in deterring animals due to habituation by some species like the finless porpoises (after an 8-month period^63^) or the harbour porpoises (after a 10-day period^71^). The response to pingers seems to depend on many factors, particularly the dolphin species on target^72^. Our study did not find evidence to support either of these caveats (Fig. 5). The treatment phase continued for 18 days and during this entire period, there was no increase in dolphin presence near the pingered site as compared to that of the non-pingered site. Similarly, there was no increase in dolphin presence during the treatment phase compared to that of the pre-treatment phase at the pingered CPOD, collectively suggesting no “dinner-bell” effect. We also see that after pinger removal, dolphins returned to the earlier deterred area, exemplifying our point that pingers were only temporary deterrents and had no lasting effect on the habitat use of dolphins.

Preventing depredation and cetacean bycatch is the primary role of an acoustic deterrent. However, its success as a mitigation measure depends on not only its efficacy, but also its acceptance and adoptability by local fisherfolk. This choice mainly depends on its impacts on fish catch. Several studies have shown that fish are sensitive to noise, especially anthropogenic noise^73–77^, which might potentially reduce the fish catch in nets, thereby reducing its adoption success by the fishers. Our study found that the catch per unit effort of fish was not significantly different between pingered and non-pingered nets, similar to results from the marine environment^62,78,79^. In terms of species composition, the dominant fish captured in the pingered and non-pingered nets were comparable. This indicates that the pingers are not being detrimental to the fish catch and hence, their possibility of adoption as a bycatch mitigation measure strengthens. However, due to a change in fish season towards the end of our experiment, diversity of fish catch decreased and more data from longer experiments, spanning different seasons is required to substantiate this finding.

For the widespread and long-term use of pingers, some limitations need to be addressed. Studies have shown that a depletion in the battery level of pingers can lead to the attraction of the animals rather than displacing them^17^; this needs to be field-tested in the current habitat. Likewise, the differential response of dolphins of different age-classes to pingers that we obtained in our experiment needs more investigation. While we found no effect of pingers on fish catch, the influence of season or any species-specific deterrence in fish catch due to pingers and a long-term study assessing the effect of pingers on fish catch is required to recognize this impact. There are reports of habituation when pingers are used intermittently^65^ in a particular area. For these purposes, a complete mapping of the fishing practices along with their spatial and temporal distribution is necessary to plan the field adoption of pingers. We also need to gain insight into the willingness of fishers to adopt these devices and deploy them on their fishing nets. In this context, it is important to understand whether fishers will adopt the use of pingers while compromising on monetary benefits from dolphin bycatches. The activities leading to these monetary benefits have legal consequences, and through social surveys it is understood that only few communities are involved^14^, a targeted sensitization approach would be apt in this situation to gain acceptance for use of pingers. Additionally, involvement of stakeholders who work in close association with fishing communities, like fisheries department needs to be explored. These stakeholders generally have incentives for fishers, where they work towards betterment of livelihoods. When incentives for use of pingers is managed through such channels (e.g. subsidy, development of cooperatives), the communities will be more accepting of use of pingers. Another avenue to be explored is a policy decision for making use of pingers mandatory by law, like the fishing vessels of the European waters^80^, California and Oregon regions^68^. Pingers are expensive and the optimum deployment interval to maintain that is economically feasible also needs to be arrived at. Proper implementation of the pingers with the fisheries and fisherfolk needs to be planned, as it has been difficult even for some sophisticated fisheries of developed countries^17^. Avenues like setting up local cottage industries for manufacturing pinger like devices, which can help the local economy in parallel or possibilities for government subsidies can be explored.

Our study demonstrates that pingers are by far the most promising and effective bycatch mitigation device available for Ganges River dolphins currently. These devices will help prevent net entanglement and reduce depredation rates, which has become a significant threat to the Ganges River dolphins^14,51,52^. Especially for a species such as Ganges River dolphins, where the growth rate is very slow, curbing deaths due to bycatch is of utmost importance, without which population crashes and extinctions will be quick to happen and difficult to recover from. Bycatch is incidental in most cases, and pingers acting as a preventive measure would be an asset to the existing conservation measures. It also brings in a new scope of exploring a sustainable method—conserving dolphins as well as protecting the livelihoods of the people. Integrating the use of pingers with the implementation of temporal closure areas or temporary sanctuaries to double up conservation measures can be explored—this will aid in replenishing the biodiversity while causing only a minimum change in the practices of river-dependent communities.

## Supplementary Information

Supplementary Information.

## Data Availability

All data generated or processed during this study is included in the manuscript and in the Supplementary information.
