# Supplementary material for: Pingers are effective in reducing net entanglement of river dolphins
Source: Sci Rep. 2022 Jun 7;12:9382. doi: 10.1038/s41598-022-12670-y (PMC9174236; doi:10.1038/s41598-022-12670-y)
Supplement: Supplementary file 1 — Supplementary Information. [file 41598_2022_12670_MOESM1_ESM.docx]

Supplementary material


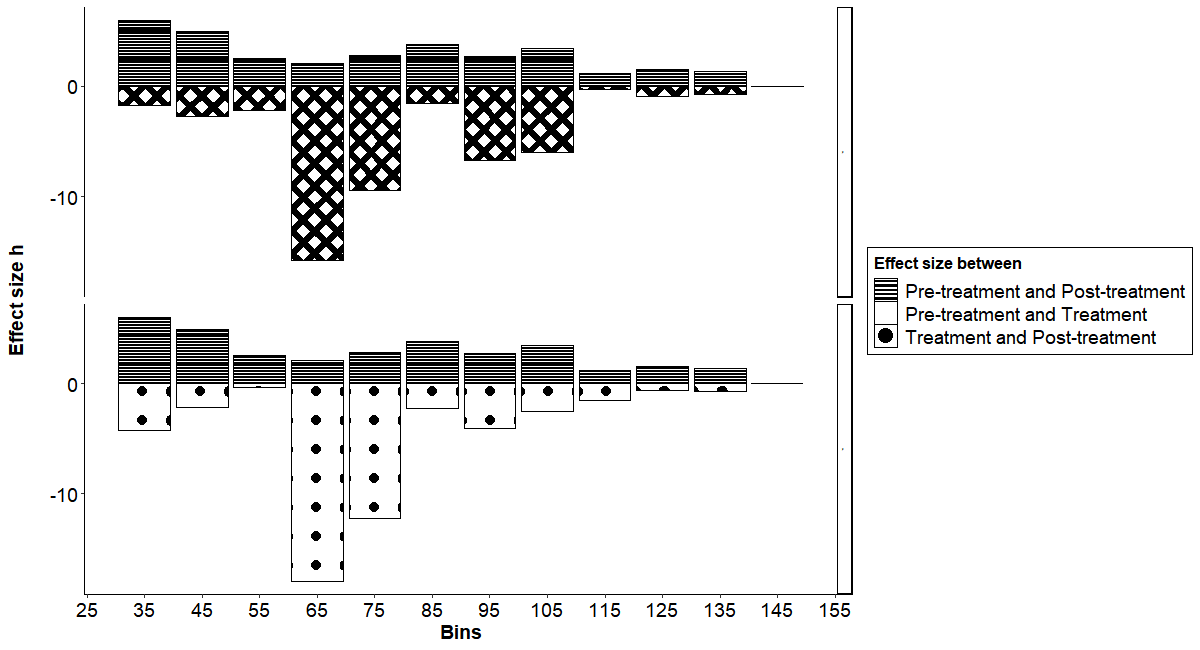


Supplementary Figure S1: Graph depicting the pairwise effect size comparison of Frequency (of dolphin clicks) in all three phases of the experiment


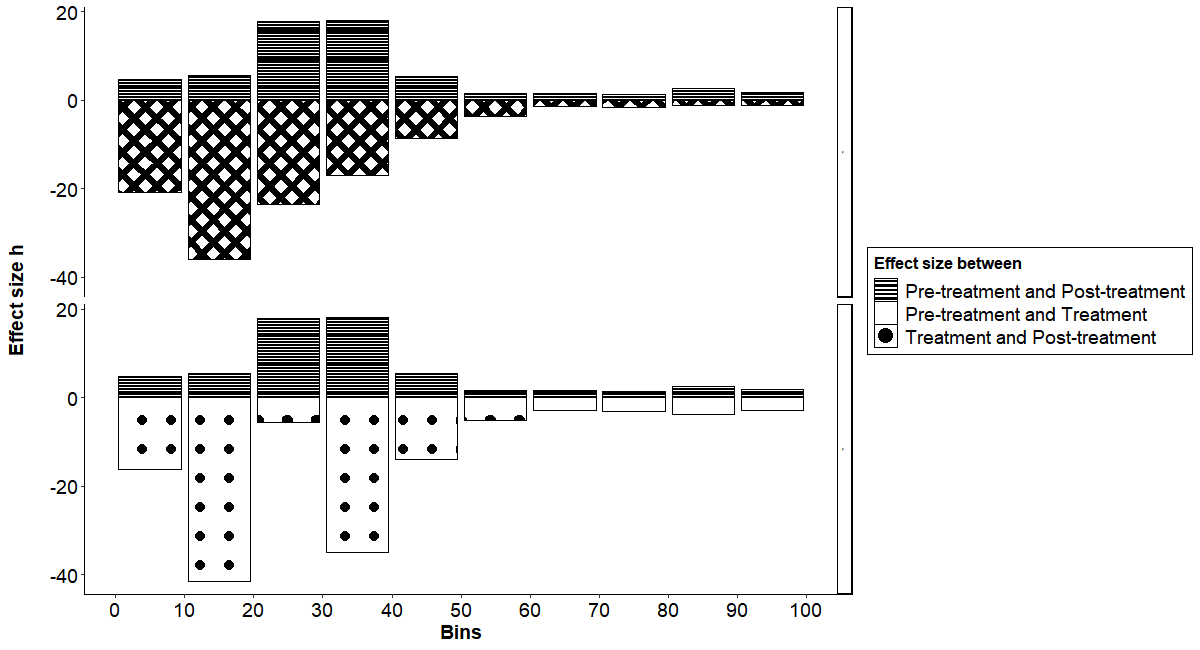


Supplementary Figure S2: Graph depicting the pairwise effect size comparison of SPL (of dolphin clicks) in all three phases of the experiment


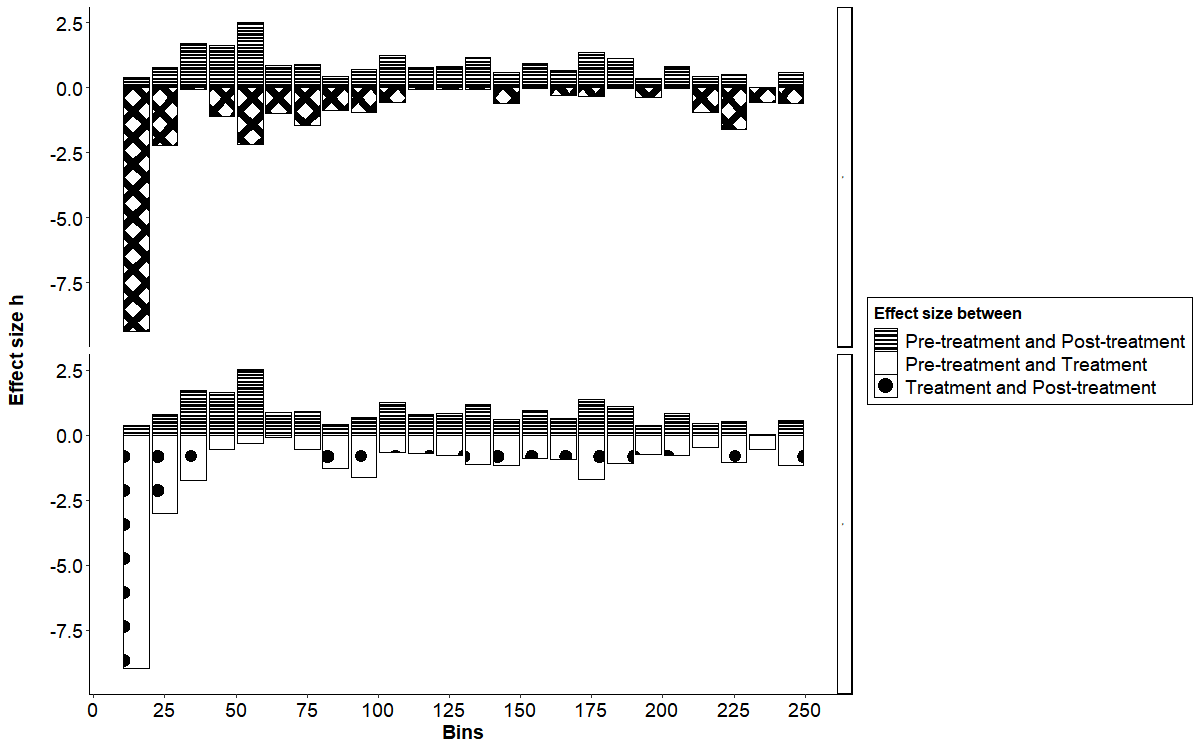


Supplementary Figure S3: Graph depicting the pairwise effect size comparison of Inte-rclick interval (of dolphin clicks) in all three phases of the experiment
